# Supplementary material for: Identification of Freezing-Responsive microRNAs and Their Targets in Chinese Jujube by Small RNA and Degradome Sequencing
Source: Int J Mol Sci. 2026 Sep 4;27(17):7912. doi: 10.3390/ijms27177912 (PMC13566890; doi:10.3390/ijms27177912)
Supplement: Supplementary file 1 [file ijms-27-07912-s001.zip › Supplementary Material S2/novel_miR_53.pdf]

Provisional ID	: novel_miR_53
Score total	: 0.4
Score for star read(s)	: -1.3
Score for read counts	: 0
Score for mfe	: 0.1
Score for randfold	: 1.6
Score for cons. seed	:
Total read count	: 594
Mature read count	: 594
Loop read count	: 0
Star read count	: 0

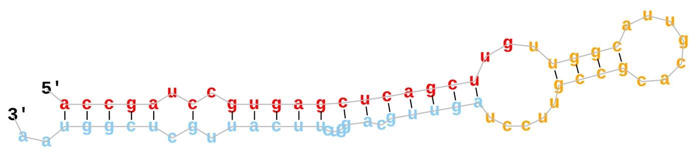

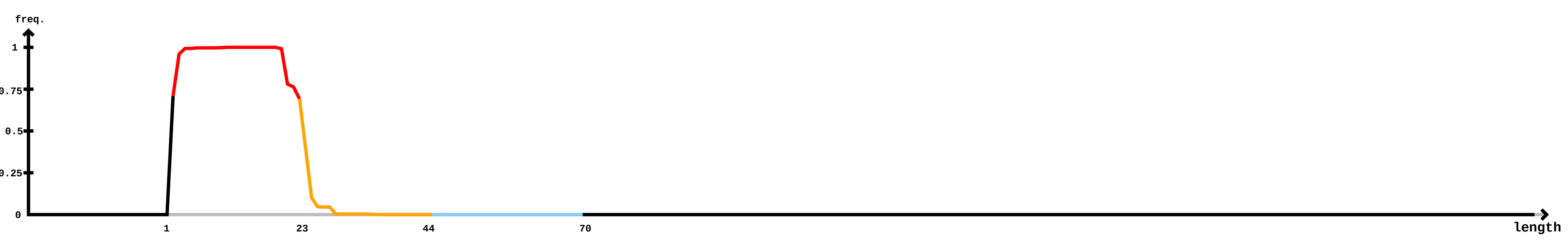

Mature Star

5'- uuguucauuuaguaacguauuuaccgauccgugagcucagcuuguuggcauugcacgccguuccuaguugcaggucuucauugcucgguaaaccaaaaaauagcaauguauuuuauuaauuuuauuugauaaaauuuauuaauaucacuuuugaugagacauaaauuaaacaagucuauuuaauuagauuuuugacaauaaauugucaauuaauuaacuuuugacaacuugcauauauuugucguuguac -3' exp

(((((.(((((((..(((.((((((((.(.((((((((((((...((((......)))).....))))).))...))))).).)))))))).(((((.(((..((((.((((((((((......)))))))))).))))..)))...))))))))..)).)))))..)))))(((((......)))))..(((((((....))))))).............(((((..(((......))).)))))..	reads	mm	sample
......................accgauccgugagcucag..	1	0	LT-2
......................accgauccgugagcucag..	3	0	LT-3
......................accgauccgugagcucag..	1	0	LT-1
......................accgauccgugagcucagc...	4	0	JT-2
......................accgauccgugagcucagc...	6	0	LT-1
......................accgauccgugagcucagc...	9	0	LT-3
......................accgauccgugagcucagc...	6	0	JT-3
......................accgauccgugagcucagc...	6	0	LT-2
......................accgauccgugagcucagc...	15	0	JCK-2
......................accgauccgugagcucagc...	5	0	JCK-1
......................accgauccgugagcucagc...	2	0	LCK-3
......................accgauccgugagcucagc...	20	0	JT-1
......................aNcgauccgugagcucagc...	1	1	JCK-2
......................accgauccgugagcucagc...	6	0	JCK-3
......................accgauccgugagcucagcu..	2	0	LT-1
......................accgauccgugaCcucagcu..	1	1	LT-2
......................accgauccgugagcucagcu..	3	0	LT-3
......................accgauccgugagcucagcu..	3	0	LT-2
......................accgauccgugagcucagcuu...	9	0	JCK-2
......................accgauccgugagcucagcuu...	2	0	LT-3
......................accgauccgugagcucagcuu...	9	0	JCK-3
......................accAauccgugagcucagcuu...	1	1	JCK-3
......................accgauccgugagcucagcuu...	9	0	JCK-1
......................accgauccgugagcucagcuu...	3	0	LT-2
......................accgauccgugagcucagcuu...	3	0	LT-1
......................accgauccgugagcucagcuug..	37	0	LCK-1
......................accgauccgugagcucCgcuug..	2	1	LCK-2
......................accgauccgugagcucagcuug..	61	0	JCK-2
......................accgauccgugagcucagcuug..	10	0	LT-2
......................Cccgauccgugagcucagcuug..	1	1	LCK-1
......................accgauccgugagcucagcuuU..	2	1	JCK-3
......................accgauccgugagcucagcuug..	4	0	JT-3
......................accgauccgugagcucagcuug..	28	0	JCK-1
......................accgauccgugagcucagcuuU..	1	1	LT-1

```
                      Mature                                     Star
uuguucauuuaguaacguauuuaccgauccgugagcucagcuuguuggcauugcacgccguuccuaguugcaggucuucauugcucgguaaaccaaaaaauagcaauguauuuuauuaauuuuauuugauaaaauuuauuaauaucacuuuugaugagacauaaauuaaacaagucuauuuaauuagauuuuugacaauaaauugucaauuaauuaacuuuugacaacuugcauauauuugucguuguac
......................Cccgauccgugagcucagcuug..............................................................................................................................................................................................................   1   1   LT-3
......................accgauccgugaNcucagcuug..............................................................................................................................................................................................................   1   1   LCK-1
......................accgauccgugagcucagcuuU..............................................................................................................................................................................................................   4   1   JCK-2
......................accgaucUgugagcucagcuug..............................................................................................................................................................................................................   2   1   JCK-1
......................accgauccgugagcucagcuuC..............................................................................................................................................................................................................   1   1   JCK-2
......................accgauccgugagcucagcuug..............................................................................................................................................................................................................   9   0   LT-3
......................accgauccgugagcucCgcuug..............................................................................................................................................................................................................   2   1   LCK-1
......................accgauccgugagcucagcuug..............................................................................................................................................................................................................   3   0   JT-2
......................accgauccgugagcucagcuuU..............................................................................................................................................................................................................   3   1   JCK-1
......................accgauccgugagcucagcuug..............................................................................................................................................................................................................   7   0   LT-1
......................accgCuccgugagcucagcuug..............................................................................................................................................................................................................   1   1   LCK-1
......................acNgauccgugagcucagcuug..............................................................................................................................................................................................................   1   1   LT-2
......................accgauccgugagcucagcuug..............................................................................................................................................................................................................   10  0   JT-1
......................accgauccgugagcucagcuug..............................................................................................................................................................................................................   26  0   LCK-2
......................accgauccgugagcucagcuug..............................................................................................................................................................................................................   26  0   JCK-3
......................accgauccgugagcucagcuugu.............................................................................................................................................................................................................   2   0   JCK-3
......................aNcgauccgugagcucagcuugu.............................................................................................................................................................................................................   1   1   JCK-3
......................accgauccgugagcucagcuugu.............................................................................................................................................................................................................   4   0   JCK-2
......................accgauccgugagcucagcuugu.............................................................................................................................................................................................................   4   0   LT-3
......................accgauccgugagcucagcuugu.............................................................................................................................................................................................................   7   0   LCK-3
......................accgauccgugagcucagcuugu.............................................................................................................................................................................................................   1   0   JCK-1
......................accgauccgugagcucagcuuguA............................................................................................................................................................................................................   1   1   JCK-3
......................accgauccgugagcucagcuuguu............................................................................................................................................................................................................   2   0   JCK-3
......................accgauccgugagcucagcuuguA............................................................................................................................................................................................................   1   1   JCK-1
......................accgauccgugagcucagcuuguC............................................................................................................................................................................................................   2   1   JCK-3
......................accgauccgugagcucagcuuguu............................................................................................................................................................................................................   5   0   LT-2
......................accgauccgugagcucagcuuguA............................................................................................................................................................................................................   2   1   JCK-2
......................accgauccgugagcucagcuuguA............................................................................................................................................................................................................   1   1   LT-1
......................accgauccgugagcucagcuuguu............................................................................................................................................................................................................   9   0   JCK-1
......................accgauccgugagcucagcuuguu............................................................................................................................................................................................................   6   0   JCK-2
......................accgauccgugagcucagcuuguug...........................................................................................................................................................................................................   1   0   LT-3
......................accgauccgugagcucagcuuguuggc.........................................................................................................................................................................................................   1   0   LT-1
.......................ccgauccgugagcucagc.................................................................................................................................................................................................................   3   0   LT-2
.......................ccgauccgugagcucagc.................................................................................................................................................................................................................   2   0   LCK-3
.......................ccgauccgugagcucagc.................................................................................................................................................................................................................   4   0   LCK-1
.......................ccgauccgugagcucagc.................................................................................................................................................................................................................   4   0   JT-3
.......................ccgauccgugagcucagc.................................................................................................................................................................................................................   6   0   LT-3
.......................ccgauccgugagcucagc.................................................................................................................................................................................................................   2   0   LCK-2
.......................ccgauccgugagcucagc.................................................................................................................................................................................................................   5   0   JCK-1
.......................ccgauccgugagcucagc.................................................................................................................................................................................................................   17  0   JT-1
.......................ccgauccgugagcucagc.................................................................................................................................................................................................................   2   0   LT-1
.......................cNgauccgugagcucagc.................................................................................................................................................................................................................   1   1   JT-1
.......................ccgauccgugagcucagcuu...............................................................................................................................................................................................................   1   0   JCK-3
.......................ccgauccgugagcucagcuu...............................................................................................................................................................................................................   2   0   LT-2
.......................ccgauccgugagcucagcuu...............................................................................................................................................................................................................   1   0   JCK-1
.......................ccgauccgugagcucagcuuC..............................................................................................................................................................................................................   2   1   JCK-2
.......................ccgauccgugagcucagcuug..............................................................................................................................................................................................................   3   0   JCK-3
.......................ccgauccgugagcucagcuug..............................................................................................................................................................................................................   22  0   LCK-1
.......................ccgauccgugagcucagcuug..............................................................................................................................................................................................................   26  0   LCK-2
.......................ccgauccgugagcucagcuug..............................................................................................................................................................................................................   6   0   JCK-1
.......................ccgauccgugagcucagcuuC..............................................................................................................................................................................................................   1   1   JCK-3
.......................ccgauccgugagcucagcuug..............................................................................................................................................................................................................   4   0   JT-3
.......................ccgauccgugagcucagcuuA..............................................................................................................................................................................................................   1   1   JCK-2
.......................ccgauccgugagcucagcuug..............................................................................................................................................................................................................   3   0   LT-2
.......................ccgauccgugagcucagcuug..............................................................................................................................................................................................................   1   0   JT-2
.......................ccgauccgugagcucagcuug..............................................................................................................................................................................................................   5   0   JCK-2
.......................ccgauccgugagcucagcuugu.............................................................................................................................................................................................................   1   0   JCK-2
.......................ccgauccgugagcucagcuuguuggc.........................................................................................................................................................................................................   14  0   LCK-2
.......................ccgauccgugagcucagcuuguuggc.........................................................................................................................................................................................................   10  0   LCK-1
........................cgauccgugagcucagcu.................................................................................................................................................................................................................   1   0   LT-3
........................cgauccgugagcucagcuu................................................................................................................................................................................................................   3   0   LT-3
........................cgauccgugagcucagcuug...............................................................................................................................................................................................................   3   0   LCK-3
........................cgauccgugagcucagcuug...............................................................................................................................................................................................................   1   0   JCK-3
........................cgauccgugagcucagcuug...............................................................................................................................................................................................................   2   0   JCK-1
........................cgauccgugagcucagcuug...............................................................................................................................................................................................................   2   0   LT-1
........................cgauccgugagcucagcuugu..............................................................................................................................................................................................................   4   0   JCK-2
........................cgauccgugagcucagcuugu..............................................................................................................................................................................................................   1   0   JCK-3
........................cgauccgugagcucagcuuguu.............................................................................................................................................................................................................   3   0   LT-1
..........................auccgugagcucagcuugu..............................................................................................................................................................................................................   1   0   LT-3
..........................auccgugagcucagcuugu..............................................................................................................................................................................................................   1   0   LT-2
```

```
                      Mature                                          Star
uuguucauuuaguaacguauuuaccgauccgugagcucagcuuguuggcauugcacgccguuccuaguugcaggucuucauugcucgguaaaccaaaaaauagcaauguauuuuauuaauuuuauuugauaaaauuuauuaauaucacuuuugaugagacauaaauuaaacaagucuauuuaauuagauuuuugacaauaaauugucaauuaauuaacuuuugacaacuugcauauauuugucguuguac
..............................gugagcucagcuuguuggcauugca...................................................................................................................................................................................................   1   0   JCK-2
...............................ugagcucGgcuuguuggcauugcac..................................................................................................................................................................................................   1   1   LT-3
```
